# Effect of mono- and dichromatic light quality on growth rates and photosynthetic performance of *Synechococcus* sp. PCC 7002

**DOI:** 10.3389/fmicb.2014.00488

**Published:** 2014-09-19

**Authors:** Hans C. Bernstein, Allan Konopka, Matthew R. Melnicki, Eric A. Hill, Leo A. Kucek, Shuyi Zhang, Gaozhong Shen, Donald A. Bryant, Alexander S. Beliaev

**Affiliations:** ^1^Biological Sciences Division, Pacific Northwest National LaboratoryRichland, WA, USA; ^2^Chemical and Biological Signature Science, Pacific Northwest National LaboratoryRichland, WA, USA; ^3^Department of Biological Sciences, Purdue UniversityW. Lafayette, IN, USA; ^4^Department of Biochemistry and Molecular Biology, The Pennsylvania State UniversityUniversity Park, PA, USA; ^5^Department of Chemistry and Biochemistry, Montana State UniversityBozeman, MT, USA

**Keywords:** cyanobacteria, photosynthesis, chlorophyll, phycobiliprotein, turbidostat, fluorescence

## Abstract

*Synechococcus* sp. PCC 7002 was grown to steady state in optically thin turbidostat cultures under conditions for which light quantity and quality was systematically varied by modulating the output of narrow-band LEDs. Cells were provided photons absorbed primarily by chlorophyll (680 nm) or phycocyanin (630 nm) as the organism was subjected to four distinct mono- and dichromatic regimes. During cultivation with dichromatic light, growth rates were generally proportional to the total incident irradiance at values <275 μmol photons m^−2^ · s^−1^ and were not affected by the ratio of 630:680 nm wavelengths. Notably, under monochromatic light conditions, cultures exhibited similar growth rates only when they were irradiated with 630 nm light; cultures irradiated with only 680 nm light grew at rates that were 60–70% of those under other light quality regimes at equivalent irradiances. The functionality of photosystem II and associated processes such as maximum rate of photosynthetic electron transport, rate of cyclic electron flow, and rate of dark respiration generally increased as a function of growth rate. Nonetheless, some of the photophysiological parameters measured here displayed distinct patterns with respect to growth rate of cultures adapted to a single wavelength including phycobiliprotein content, which increased under severely light-limited growth conditions. Additionally, the ratio of photosystem II to photosystem I increased ~40% over the range of growth rates, although cells grown with 680 nm light only had the highest ratios. These results suggest the presence of effective mechanisms which allow acclimation of *Synechococcus* sp. PCC 7002 acclimation to different irradiance conditions.

## Introduction

Growth rate is a variable that affects the physiological state of all microbial organisms (Neidhardt et al., 1990); one obvious manifestation of this is the necessity to satisfy demands of biosynthesis and energy acquisition as growth rate increases. When growth is constrained by the availability of an essential resource, cells upregulate synthesis of the molecular machinery that acquires this limiting resource (Tempest et al., 1983; Ludwig and Bryant, 2012). To that end, photosynthetic organisms face the additional challenge of dealing with actinic light as an essential resource due to the temporal availability, quantity, and quality of photons incident to the environment. Under light limitation, photosynthetic pigment levels typically increase to facilitate energy acquisition (Macintyre et al., 2002). When light is in excess, the macromolecular composition and ultrastructure of the photosynthetic apparatus undergoes acclimation to avoid photoinhibition (Falkowski and Raven, 2013).

Despite extensive analysis of the relationship between irradiance and photosynthetic activity (Myers, 1980; Dubinsky et al., 1986; Henley, 1993; Macintyre et al., 2002), the effects of light quality and quantity on growth rate has been less systematically investigated. In part, this is due to the technical difficulty of maintaining a well-defined light climate in batch cultures for which self-shading increases with biomass density. One solution is to use continuous cultures, which attain steady state and provide well-controlled and reproducible conditions for physiological and systems biology analyses (Bull, 2010). While chemostat cultures have traditionally been used to explore microbial growth kinetics at definable steady states, analyses are constrained toward investigation of nutrient-limited physiologies (Smith, 1995; Huisman et al., 2002). Alternatively, turbidostat cultivation provides a means to investigate steady states defined by the maximum growth rate of an organism at a given condition, without altering the cell culture density of the culture. In the context of phototrophic growth, turbidostats enable the maintenance of optically thin (i.e., low OD) cultures, which significantly decrease light heterogeneity while providing tight control over environmental parameters (Melnicki et al., 2013).

In this study, we applied turbidostat cultivation to interrogate the interacting effects of varying 630- and 680-nm light intensities upon steady-state growth of a unicellular, euryhaline cyanobacterium *Synechococcus* sp. strain PCC 7002 (hereafter *Synechococcus* 7002). This organism has become a widely studied model system for investigating cyanobacterial photophysiology and metabolism, because it is capable of growth over a wide range of NaCl concentrations (0–2.0 M) and under high irradiance levels (Batterton and Van Baalen, 1971). Its tolerance to very high irradiance (Nomura et al., 2006) and resistance to reactive oxygen species have been correlated, at least in part, to very high constitutive transcript levels encoding putative protective enzymes (Ludwig and Bryant, 2012). Under optimal conditions, *Synechococcus* 7002 displays growth rates which rank among the fastest reported for any cyanobacterium. While the underpinnings of a high growth rate are not fully understood, this phenotype may partly arise from a naturally high PS II to PS I ratio (typically ~0.5) (Zhao et al., 2001), relatively high chlorophyll (Chl) content per cell, and low phycocyanin (PC) to allophycocyanin (APC) ratio (2:1) of the phycobilisome (Gomez-Lojero et al., 2003). To investigate the adaptive mechanisms of the photosynthetic systems in *Synechococcus* 7002 to changing light regimes and growth rates, we analyzed turbidostat cultures that had reached a steady state at a constant irradiance over several generations, as opposed to studying transient responses to shifts in the light climate. The growth output was complemented by spectroscopic measurements to determine the contents of pigments and photosystems, and pulse amplitude-modulated fluorometry to infer properties of PS II and subsequent downstream processes.

## Materials and methods

### Bacterial strains and growth

*Synechococcus* 7002 was grown in modified basal A medium containing: 18 g/L NaCl, 0.6 g/L KCl, 0.9 g/L NH_4_Cl, 5.0 g/L MgSO_4_·7H_2_O, 50 mg/L KH_2_PO_4_, 266 mg/L CaCl_2_, 30 mg/L Na_2_EDTA·2H_2_O, 3.89 mg/L FeCl_3_·6H_2_O, 1 g/L Tris·HCl (pH 8.2), 34.26 mg/L H_3_BO_3_, 4.32 mg/L MnCl_2_·4H_2_O, 0.315 mg/L ZnCl_2_, 0.03 mg/L MoO_3_, 12.15 μg/L CoCl_2_·6H_2_O, 3 μg/L CuSO_4_·5H_2_O, and 4 μg/L vitamin B_12_ (Stevens and Porter, 1980; Ludwig and Bryant, 2011). Starter cultures for controlled-cultivation experiments were inoculated from frozen stocks and were grown as batch cultures in sealed serum bottles initially flushed with 95–5% N_2_-CO_2_ gas mixture under continuous white-light irradiance at 50 μmol photons m^−2^ · s^−1^. Photobioreactors were operated at 5.5-L volume with 250 rpm agitation and maintained at 30°C, and pH 7.5 (controlled via addition of either 2M NaOH and HCl). Unless otherwise stated, cultures were maintained as light-limited with scalar incident irradiance (I_i_) ranging from 50 to 275 μmol photons ·m^−2^ · s^−1^. The photobioreactor was sparged with a gas mixture of 98% N_2_ and 2% CO_2_ at 4.1 L·min^−1^. Physiological steady-state was inferred from continuity (≤3% variation between measurements) of the following growth readouts: OD_730_, pH, and dissolved O_2_ concentration. Samples for all the analyses were taken after at least five volume changes at steady-state conditions.

### Turbidostat cultivation

Controlled cultivation was carried out using a custom-built photobioreactor (PBR) equipped with an aluminum enclosure housing light-emitting diode (LED) arrays (Melnicki et al., 2013). The LED illuminator chips (Marubeni America Corporation, New York, NY), which provided peak emissions at 630 and 680 nm, were used to preferentially excite PC and/or Chl *a*, respectively. For turbidostat operation, constant optical density (OD_730_) of 0.09 ± 0.01 was maintained by varying influent/effluent pump speed using the New Brunswick Bioflo 310 fermenter platform (Eppendorf, Inc., Enfield, CT). Incident and transmitted irradiance was measured with 6 opposing 2π quantum sensors (LI-210SA Photometric Sensor, LI-COR Biosciences, Lincoln, NE) and intercalibrated with a 4π submerged quantum sensor (LI-193SA Spherical Underwater Quantum Sensor, LI-COR Biosciences). Hence, scalar incident irradiance (I_i_) is reported here as quanta incident to the center of the reactor and has been confirmed to be both axially and radially isotropic. Steady-state were controlled through set-points determined by intercalibrating optical density (OD_730_) and transmitted light. The lowest set point of the 630 nm LED (~50 μmol photons ·m^−2^ · s^−1^) was determined to be very near the limit of accuracy required to deliver constant, monochromatic incident irradiance. Steady-state specific growth rates were not determined for monochromatic growth above I_i_ = 120 μmol photons ·m^−2^ · s^−1^; hence only two conditions were considered for 630 nm monochromatic growth.

### Analytical procedures

Cell dry weight concentrations were measured directly as ash- free dry weight (AFDW) (Pinchuk et al., 2010) and compared in a standard curve to the indirect OD_730_ measurements obtained using a Genesys 20 visible spectrophotometer (Thermo Scientific, Rockford, IL). Protein was quantitated using BCA protein reagent (Thermo Scientific). Chl *a* and phycocyanin concentrations were estimated using a previously described method that corrects the effect of scatter on absorbance from whole-cell suspensions (see Supplemental Methodologies) (Myers, 1980; Burns et al., 2006). Dissolved O_2_ concentration in the reactor was measured with a Clark O_2_ electrode (InPro^®^ 6800Series, Mettler Toledo International Inc., Columbus, OH). O_2_ production rates as a function of “white light” irradiance (tungsten incandescent) were measured inside an oxygraph chamber (Hansatech, Norfolk, UK).

### 77K fluorescence emission spectroscopy

Low-temperature fluorescence emission spectra were measured using an SLM8000-based spectrofluorometer modified for computerized, solid-state operation by On-Line Instrument Systems Inc. (Bogart, GA) as described previously (Shen et al., 2008). Cells were adjusted to equal final concentration (OD_730_ = 0.5) using 50 mM HEPES/NaOH, pH 7.0 containing 60% (v/v) glycerol, incubated 5 min in the dark and immediately frozen in liquid nitrogen. The excitation wavelength was set at 440 nm to selectively excite chlorophylls associated with photosystem II and I. Alternatively, fluorescence emission from the phycobilisomes in whole cells was selectively excited with a wavelength of 590 nm for primary excitation of phycocyanin. A 600 nm cut-on filter was used for protection of the photomultiplier. For each sample, the presented fluorescence emission spectrum is the average of four measured spectra.

### Pulse-amplitude modulated fluorometry

Photosynthetic activities of photosystem II were measured in samples obtained from steady-state turbidostat cultures using pulse amplitude-modulated fluorometry (PAM) in a DUAL-PAM-100 equipped with a photodiode detector and RG665 filter (Walz GmbH, Effeltrich, Germany). Red measuring light (620 nm) was pulsed at the lowest power at 1000 Hz in the dark and at 10,000 Hz during actinic illumination at 98 μmol photons m^−2^ · s^−1^ with 635 nm light. Fluorescence induction (FI) was measured after 15 s of darkness during a 30-s period of actinic illumination. Variable fluorescence observed during the O-J-I-P-S induction (Strasser et al., 2004; Stirbet and Govindjee, 2011) provided the basis to compare changes in the electron transport processes downstream of PS II (see Figure S1). This was specifically analyzed here as Chl fluorescence quenching from “P” to “S” states (P >> S quenching) which was used to compare the relative magnitude of reactions downstream from PS II that consume reductant. Transient fluorescence changes were measured through: (*i*) a 200-ms saturating pulse (2000 μmol photons m^−2^ · s^−1^), (*ii*) 5 s of only far-red light (730 nm), (*iii*) another 15 s of actinic light and (*iv*) 30 s darkness. The post-illumination fluorescence rise during step *iv* (see Figure S1) results from reduction of plastoquinone (PQ) from NAD(P)H or other reductants accumulated during illumination and the positive slope of fluorescence with respect to time (df/dt) was interpreted as a proxy for the rate of cyclic electron transport (CEF) (Deng et al., 2003). Similarly, the subsequent decay of post-illumination fluorescence was attributed to the re-oxidation PQ pool in the dark and was measured as df/dt 10–20 s after the maximum fluorescence signal measured in the dark (Ryu et al., 2003). Calculations for determining the relative electron transport rates (rETR = PAR · ΔF/F′_m_) have been previously described (Schreiber, 2004; Sukenik et al., 2009). The maximum values (rETR_max_) were determined from rapid light curves generated by evaluating rETR as a function of increasing PAR values (1 min step intervals).

### Measurement of the P700 content in cells

Cells were collected by centrifugation and resuspended (OD_730_ = 0.5) in 50 mM Tris-HCl, pH 8.3 buffer. The absorbance changes at 700 nm were monitored by a commercial LED pump-probe spectrometer Model JTS-10 (Bio-Logic, France). Actinic illumination was provided by a high-power (940 μmol photons ·m^−2^ · s^−1^) red LED (680 ± 50 nm), and samples were subjected to continuous illumination until maximum bleaching was achieved and P700 were assumed to be oxidized. A white LED, filtered through a 700 nm interference filter, was used to generate the measuring pulses (Edmund Optics, Inc.). PS I concentrations (c) were calculated based on absorbance change of P700 using the formula *c* = 0.46·(dI/I)/ε; where ε is the extinction coefficient (61,000 M^−1^ cm^−1^) of PS I in cyanobacteria (Witt et al., 2003). The amount of PS I per unit biomass determined using the experimentally-determined relationship of 335 mg ash free dry weight per unit OD_730_.

## Results

**Figure 1:**
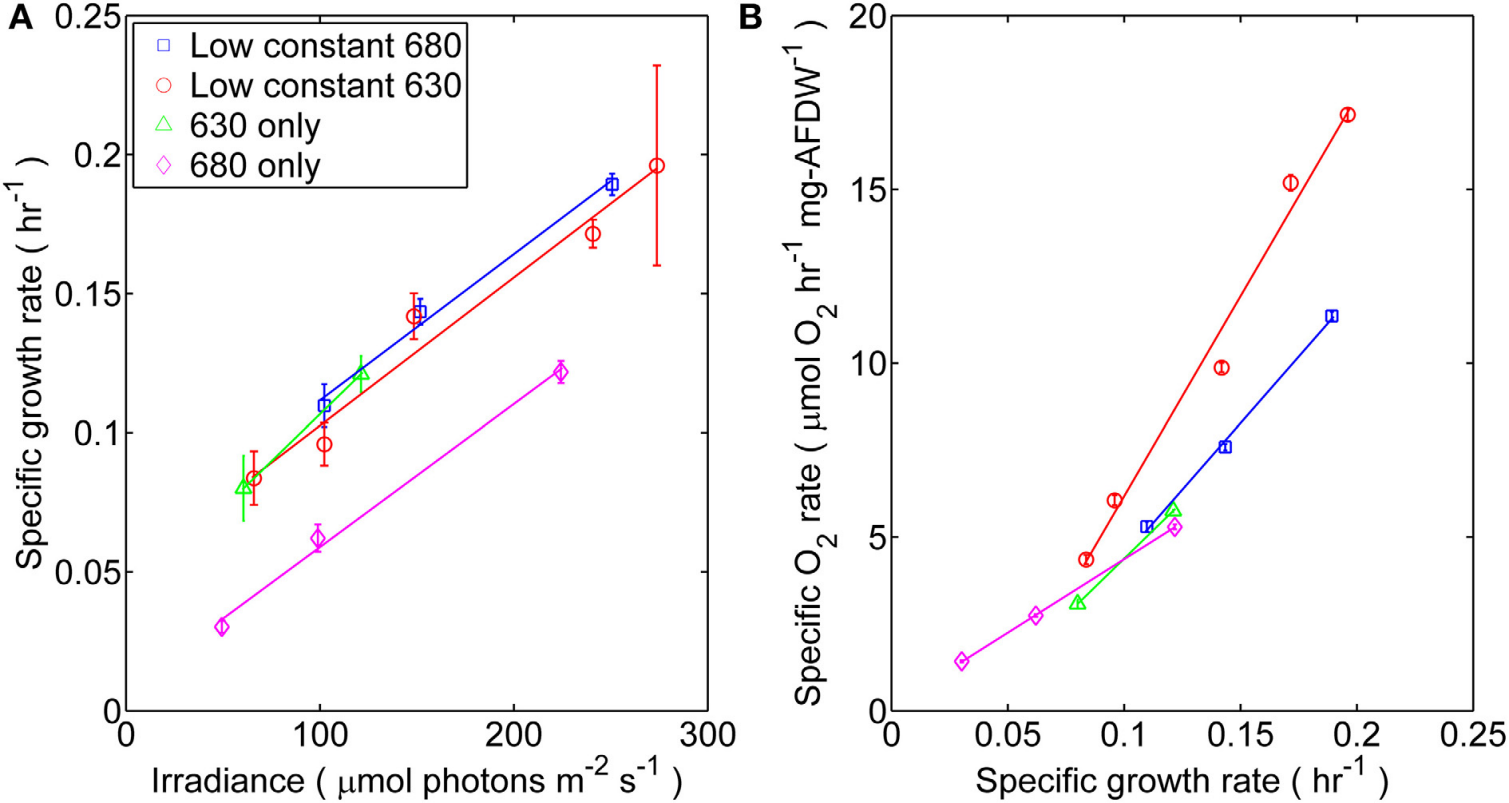
***Synechococcus* 7002 was cultured under four distinct light regimes each corresponding to mono- or dichromatic irradiance: (□) 53 μmol photons m^−2^ · s^−1^ of 680 nm light plus variable amounts of 630 nm light, (◦) 50 μmol photons m^−2^ · s^−1^ of 630 nm light plus variable amounts of 680 nm light, (Δ) 630 nm light only, or (◊) 680 nm light only. (A)** Steady-state specific growth rate measured as a function of scalar incident irradiance for *Synechococcus* 7002 turbidostat cultures. Data represents the mean from *n* > 300 data points collected through steady-state conditions; error bars represent ± 1 standard deviation. The slopes calculated from linear regression of the μ vs. I_i_ curve were (10^−7^ m^2^ μmol photons^−1^): 1.46 (53 μmol photons m^−2^ · s^−1^ of 680 nm plus variable of 630 nm), 1.48 (53 μmol photons m^−2^ · s^−1^ of 630 nm plus variable of 680 nm), 1.43 (680 nm only), and 1.88 (630 only; regression of only two data points). **(B)** The net flux of O_2_ (μmol O_2_ h^−1^ · mg^−1^_AFDW_) in turbidostat cultures, expressed as a function of specific growth rate.

Rapid specific growth rates (μ) of up to 0.2 h^−1^ (doubling time of 3.5 h) were achieved when *Synechococcus* 7002 was cultivated in turbidostat mode under light-limited conditions at 30°C (Figure 1A). Cell densities of 0.09 (OD_730_), corresponding to 34 ± 4 mg_AFDW_L^−1^, were constantly maintained to reduce self-shading in the photobioreactor. Hence, the effect of growth as a function of spectral irradiance was only imposed upon the dilution rate of the system and not on the biomass concentration. The ratio of quanta composing the mono- and dichromatic illumination of the photobioreactor was controlled by the LEDs output and consisted of four distinct experimental regimes including: (*i*) low and constant (50 μmol photons m^−2^ · s^−1^) 630 nm and variable 680 nm irradiance, (*ii*) low and constant (53 μmol photons m^−2^ · s^−1^) 680 nm and variable 630 nm irradiance, (*iii*) monochromatic 630 nm irradiance, and (*iv*) monochromatic 680 nm irradiance.

Physiological light limitation in the steady-state turbidostat cultures of *Synechococcus* 7002 was evinced by the observation that growth and net O_2_ evolution responded linearly (i.e., first-order response) with increasing scalar incident irradiance (I_i_) (Figures 1A,B). The specific growth rate increased linearly at I_i_ ≤ 275 μmol photons m^−2^ · s^−1^ but began to asymptotically approach a maximum value (0.2 h^−1^) at irradiances above this value (data not shown). The growth rates of cells irradiated with 680 nm light alone differed, and were distinctly lower by 50–70% than those of cells grown under any combination of 630 and 680 nm or 630 nm light only. Net volumetric O_2_ evolution rates, which are a surrogate for the net rate of oxygenic photosynthesis (Bernstein et al., 2014), increased linearly with growth rate (Figure 1B). However, the net O_2_ evolution rate was substantially higher for cultures irradiated with low amounts of 630 nm light than for cultures exposed to identical I_i_ values for which the intensity of 680 nm light was held low.

**Figure 2:**
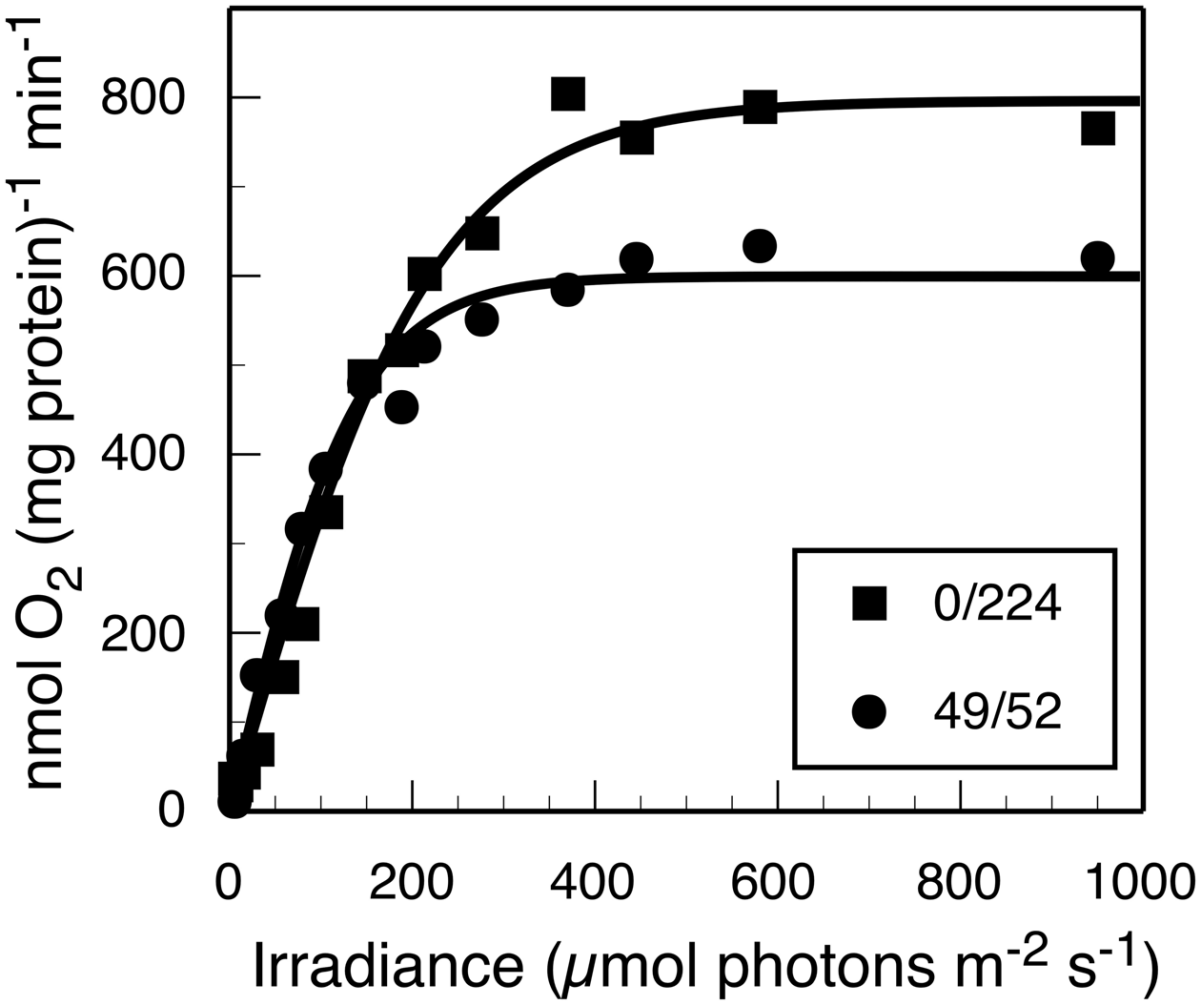
**Net rate of photosynthetic O_2_ evolution as a function of white light irradiance (PAR) for steady-state cultures sampled from the turbidostat with (■) 224 μmol photons m^−2^ · s^−1^ of 680 nm light only or (●) 49 and 52 μmol photons m^−2^ · s^−1^ of 630 and 680 nm light, respectively**. The measurements were initiated within 5 min of reactor sampling.

Furthermore, photosynthetic growth was similar across all light regimes as demonstrated by similarities between slopes of the μ vs. I_i_ relationship. These slopes are an indicator for the quantum yield of biomass production, and only account for the proportionality between the total rate of incident quanta absorbed by cellular pigments and I_i_(Kirk, 1994; Falkowski and Raven, 2013). Thus, despite the observed similarities in the quantum yields of biomass, growth rate was lower when no 630 nm light was present, presumably because phycobilisomes were not passing excitation to PS II nor able to balance the light inputs between the two photosystems (Watanabe et al., 2014). The results from oxygraph measurements, which reflect the capacity for oxygenic photosynthesis, are consistent with this interpretation (Figure 2). When provided white light, the *Synechococcus* 7002 cells grown under monochromatic 680 nm light are photosynthetically competent and able to acquire the energy needed to support the observed maximal growth rate but constrained by the absence of 630 nm irradiance in the photobioreactor.

**Figure 3:**
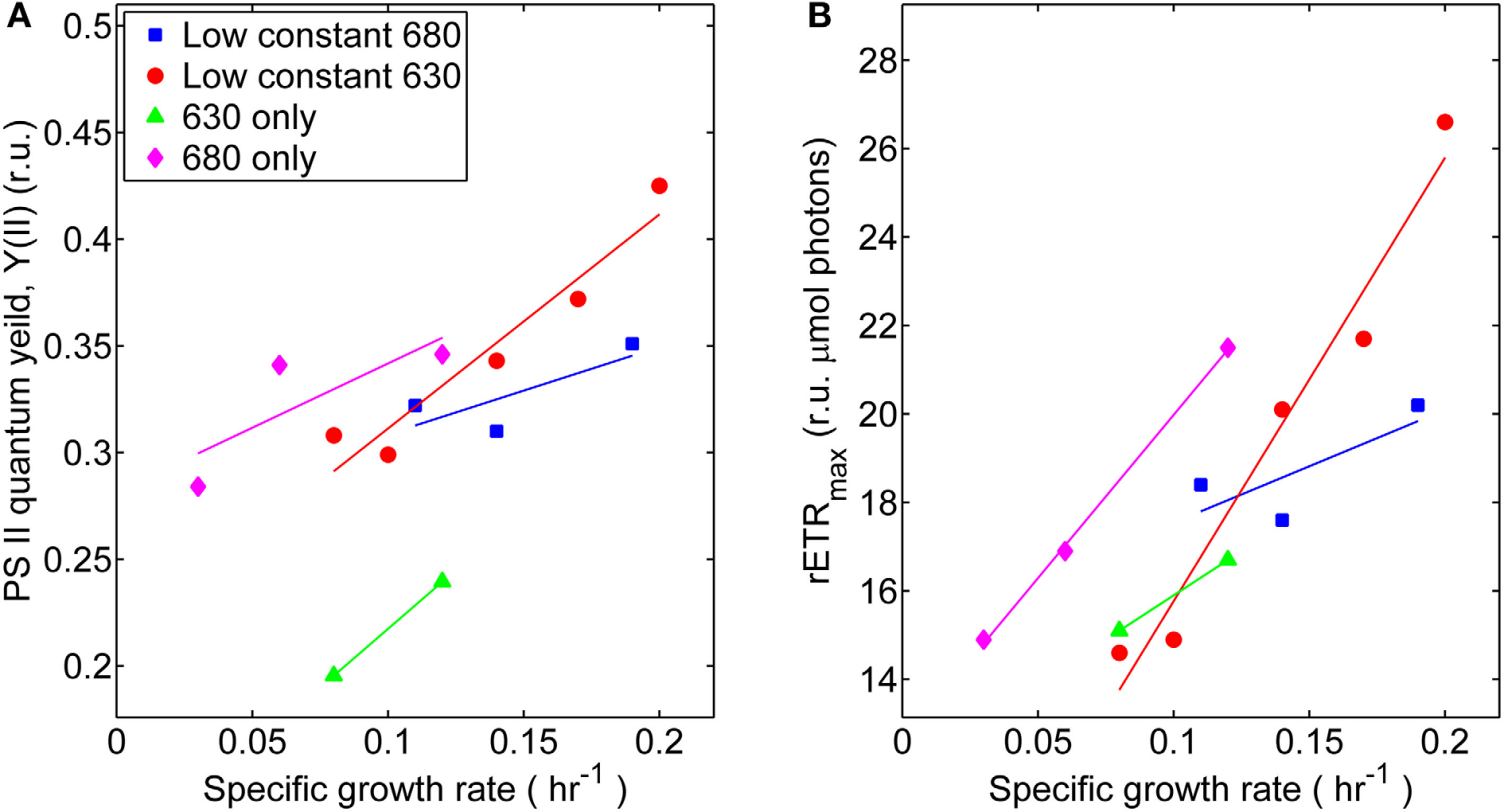
**Photosynthetic parameters derived from variable chlorophyll fluorescence measurements performed on samples harvested during turbidostat controlled steady-state at each respective light regime: (■) 53 μmol photons m^−2^ · s^−1^ of 680 nm light plus variable amounts of 630 nm light, (●) 50 μmol photons m^−2^ · s^−1^ of 630 nm light plus variable amounts of 680 nm light, (▲) 630 nm light only, or (♦) 680 nm light only. (A)** Comparisons of effective PS II quantum yields (Y_II_, given in relative units, r.u.) at different growth rates. **(B)** Comparisons of maximum relative electron transport rates (rETR_max_) at different growth rates.

The coordinated operation of the photosynthetic apparatus in *Synechococcus* 7002 at different growth rates, associated with distinct irradiance regimes, was interrogated from the kinetics of variable Chl fluorescence, fluorescence induction, and post-illumination fluorescence. In general, the photophysiological measurements associated with PS II activity tended to increase with growth rate, although the slope was shallow for the treatment series with low, constant 680 nm light and varying 630 nm irradiance (Figure 3). The monochromatic growth regimes showed substantial differences from each other. The growth-constrained cells cultured under monochromatic 680 nm light displayed comparatively higher PS II activity required to support the same growth rates as cells adapted to and grown under the 630 nm monochromatic regime. This result was determined by measuring photophysiological parameters specific to PS II and included maximum relative rate of electron transport (rETR_max_) and the effective PS II quantum yield (Y_II_). When compared at identical specific growth rates (μ = 0.12 h^−1^), the rETR_max_ and Y_II_ values were 25 and 30% lower, respectively, for cultures irradiated with 630 nm compared to 680 nm monochromatic light. Hence, cells growing in the absence of 630 nm light likely require more net energy input to support equivalent growth when compared to cells growing in the absence of 680 nm light. Similar to the oxygraph analyses, the comparisons of variable Chl measurements only indicate photosynthetic potential and not the *in situ* physiological rates or yields within the turbidostat environment.

**Figure 4:**
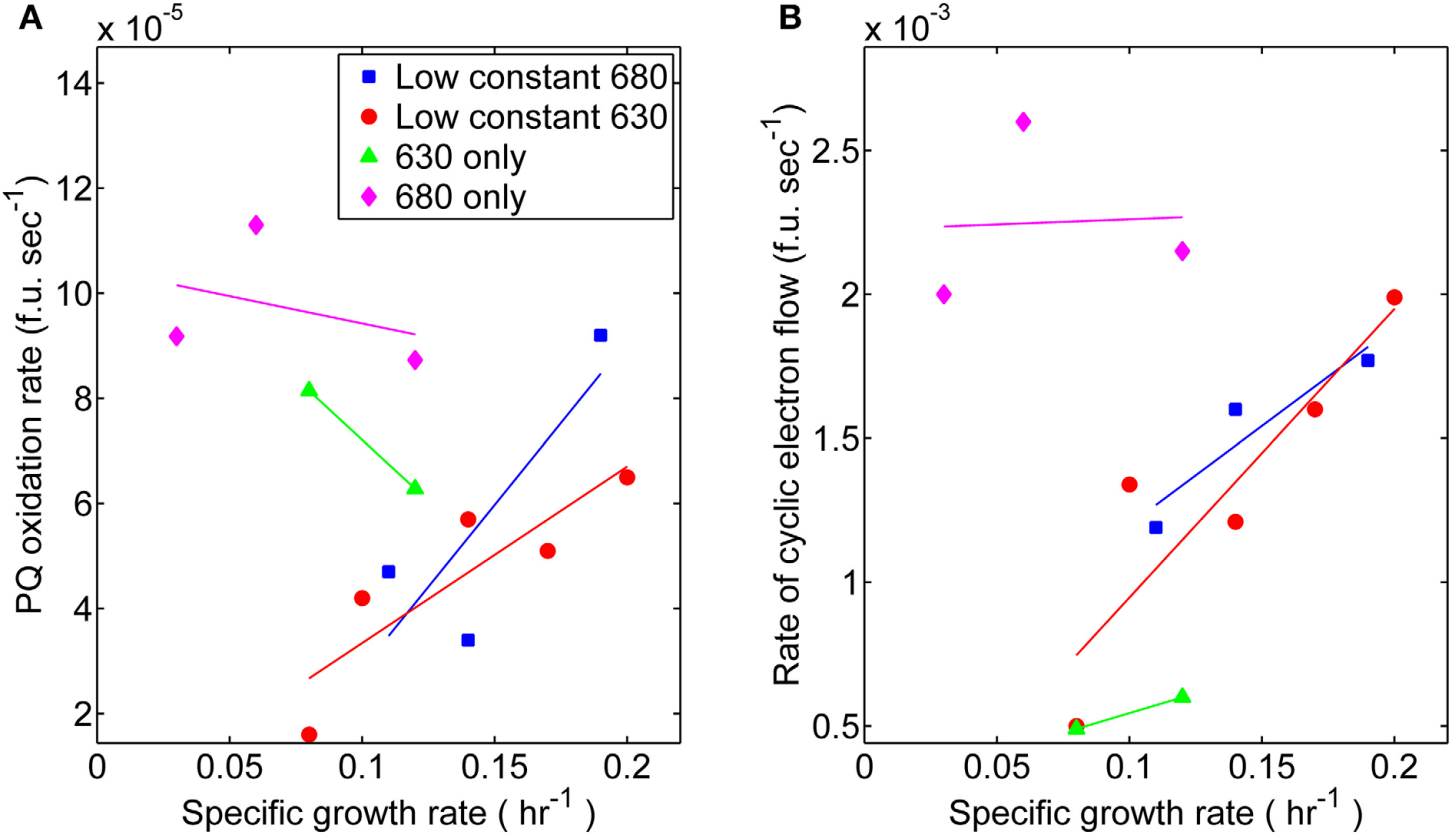
**Photosynthetic parameters related to elements of photosynthesis downstream from PS II measured from steady-state cultures corresponding toeach respective light regime: (■) 53 μmol photons m^−2^ · s^−1^ of 680 nm light plus variable amounts of 630 nm light, (●) 50 μmol photons m^−2^ · s^−1^ of 630 nm light plus variable amounts of 680 nm light, (▲) 630 nm light only, or (♦) 680 nm light only. (A)** Comparisons for the proxy rates of plastoquinone oxidation [fluorescent units (f.u.; measured in voltage) per second] in the dark measured for different growth rates. **(B)** Comparisons of proxy rates for cyclic electron flow (f.u. s^−1^) measured for different growth rates.

The relationship between growth and associated electron transport components downstream from PS II also exhibited variations with respect to monochromatic and dichromatic irradiance. These observations were made by analyzing the rates of post-illumination Chl fluorescence rise and quenching known to result, in part, from cyclic electron flow (CEF) around PS I and respiration processes resulting in “dark” PQ oxidation (Figure 4). The dark PQ oxidation rate increased linearly with growth rate when both Chl and PC were illuminated. However, when cultures were grown under either monochromatic regime, the values were significantly higher (Figure 4A). Similarly, an increasing relationship between CEF and growth rate was observed to be more pronounced during dichromatic growth compared to each respective monochromatic condition, and was highest and lowest for the 680 and 630 nm monochromatic growth conditions, respectively (Figure 4B).

**Figure 5:**
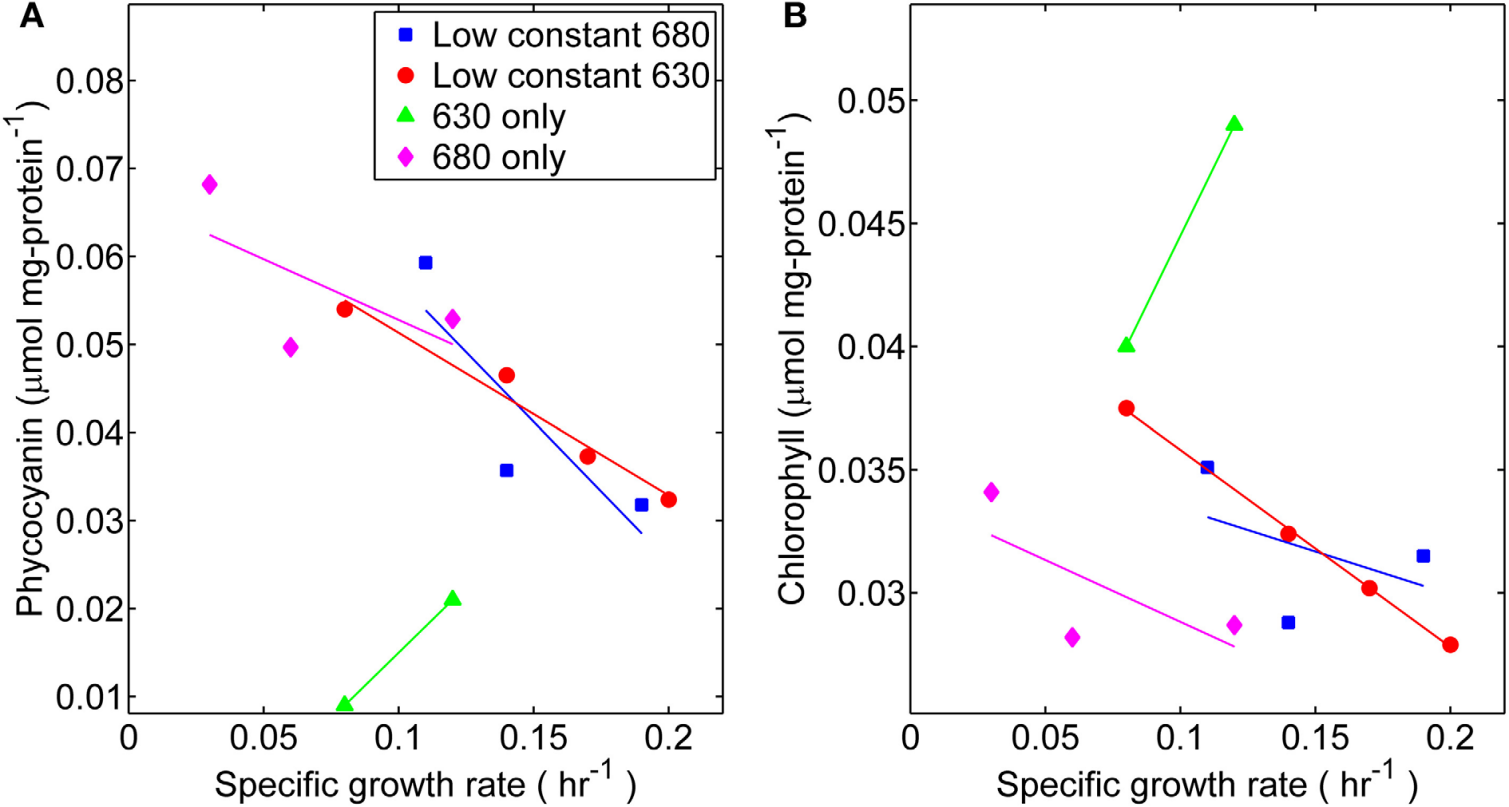
**Comparisons of (A) phycocyanin content and (B) chlorophyll content (μmol per mg protein) at different specific growth rates**. Samples were harvested directly from the turbidostat during steady-state growth corresponding to each light regime: (■) 53 μmol photons m^−2^ · s^−1^ of 680 nm light plus variable amounts of 630 nm light, (●) 50 μmol photons m^−2^ · s^−1^ of 630 nm light plus variable amounts of 680 nm light, (▲) 630 nm light only, or (♦) 680 nm light only.

Changes in pigment content were also noted as the photosynthetic apparatus of *Synechococcus* 7002 acclimated to growth under each light regime. The phycobiliprotein content of turbidostat cultures generally declined with increasing growth rate (Figure 5A). Nonetheless, cells grown under 680 nm monochromatic light, a wavelength that is not optimal for phycobiliprotein absorbance, still synthesized phycobiliproteins and yielded the highest observed phycobiliprotein to protein ratio at the lowest experimental growth rate tested in the current study. This is consistent with results from a variety of other phototrophic organisms in which the amounts of antenna pigments were inversely related to the severity of light limitation (Melis, 2009; Kwon et al., 2013). In contrast, the changes in Chl *a* content (Chl *a* to protein ratio) were less dynamic (see Table S1) over the range of growth rates tested (Figure 5B).

**Figure 6:**
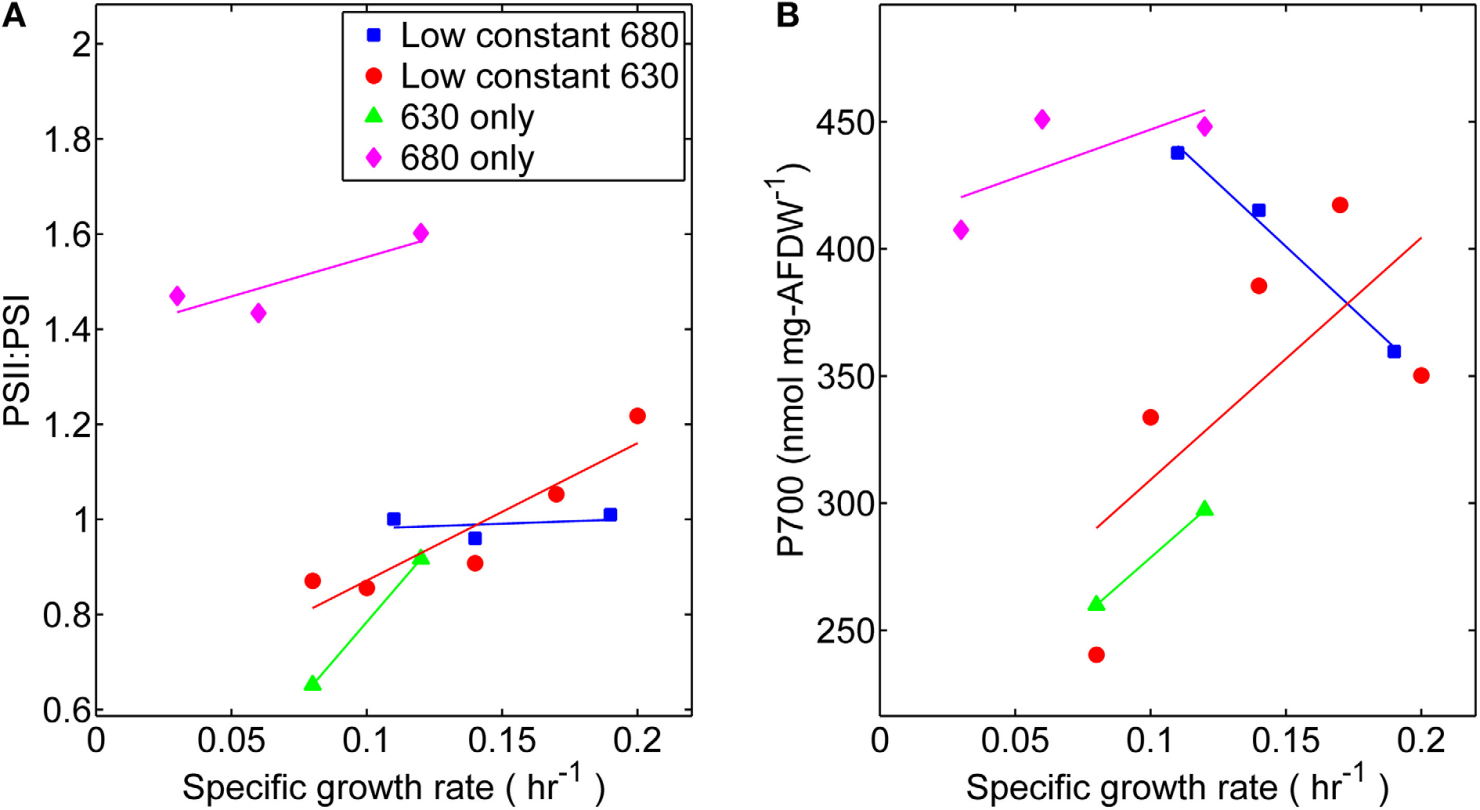
**Comparisons of (A) Photosystem II to Photosystem I ratios and (B) Photosystem I content per cell dry weight**. Samples were harvested directly from the turbidostat during steady-state growth corresponding to each light regime: (■) 53 μmol photons m^−2^ · s^−1^ of 680 nm light plus variable amounts of 630 nm light, (●) 50 μmol photons m^−2^ · s^−1^ of 630 nm light plus variable amounts of 680 nm light, (▲) 630 nm light only, or (♦) 680 nm light only.

Photophysiological changes measured at different specific growth rates were also obtained by analyzing the reaction center stoichiometry during each light experimentally controlled regime. The measured ratio of PS II to PS I reactions centers was within a relatively narrow range of 0.85–1.2, with the exception of cultures grown only under wavelengths absorbed by Chl *a* (680 nm) (Figure 6). The PS II to PS I ratio is regulated by both light intensity and spectral quality (Manodori and Melis, 1986); hence it is not surprising that the values measured were different than a previously reported value of 0.53 for *Synechococcus* 7002 (Zhao et al., 2001). Higher levels of PS II, which contains 35 Chl *a* molecules per P680, were found in these cultures compared to PS I, which contains 96 Chl *a* per P700 (Jordan et al., 2001). There was more variation of PS I content per cell, compared at each light regime, during the lower growth rates (≤0.1 h^−1^). Notably, the highest PS I contents also corresponded to those of cells grown under 680 nm monochromatic regime suggesting that content of PS II centers also increased at this condition. The latter however did not have any appreciable effect on the measured specific growth rate.

## Discussion

The current study reports growth experiments performed with optically-thin turbidostat cultures of *Synechococcus* 7002 under different monochromatic and dichromatic light regimes to test the combinatorial effects of light quality and growth rate on photophysiology. Specific growth rate was light-limited and at I_i_ of 275 μmol photons m^−2^ · s^−1^ approached a maximum of 0.20 h^−1^ (3.5 h doubling time; 30°C), which is exceptionally fast for cyanobacteria in general. While a similar 3.5 h doubling time was previously reported for *Synechococcus* 7002, these measurements were conducted at a higher temperature of 38°C using nitrate rather than ammonia as the sole N source (Sakamoto and Bryant, 2002). Accounting for temperature differences with a Q_10_ value of 2, the equivalent doubling time at 30°C would have been 6 h under the 38°C culturing condition. Other widely studied cyanobacterial strains grow significantly slower; for example, *Synechocystis* sp. PCC 6803 which was reported to have a doubling time of 12 h at (Vermass et al., 1988), and *Synechococcus elongatus* PCC 7942 with a doubling time of 6–7 h (Mori et al., 1996; Emlyn-Jones et al., 2006).

As growth rates increase in photoautotrophic cyanobacteria, the rates of reductant generation and energy acquisition from the environment must also increase to support CO_2_ fixation for subsequent biosynthesis and macromolecule polymerization. Consistent with this, the quenching of Chl fluorescence from the maximally reduced, “peak” PQ-pool state (“P”) to the quasi steady-state (“S”) state (ca. 16–24 s; see Figures S1, S2) is a consequence of reactions downstream from photosynthesis that consume reductant. This P-to-S (for P >> S) transient parameter was positively correlated to growth rate, except for the cultures grown with only 680 nm irradiance.

The physiological analyses performed on *Synechococcus* 7002 grown at defined specific growth rates are consistent with two general principles of microbial physiology: (*i*) biosynthetic and energy-generating processes increase with growth rate and (*ii*) microorganisms synthesize increased levels of molecular machinery for acquisition of a limiting resource. The limiting resource in the current study was pigment specific quanta. In particular, the cell content of phycobiliproteins in *Synechococcus* 7002 increased as growth limitation increased (decreasing μ). In contrast, the Chl *a* content (associated with the PS I and PS II reactions centers) was relatively constant across a range of growth rates that differed by nearly an order of magnitude. Previous studies on cyanobacteria invariably show an increase in phycobiliproteins under light limitation, but changes in Chl content vary from an increase that parallels the change in phycobiliproteins (Jodlowska and Latala, 2013) to instances in which more modest changes occur in Chl content (Raps et al., 1983; Kana and Glibert, 1987; Millie et al., 1990, 1992). The current study implicates the spectral quality (i.e., wavelength distribution) of incident irradiance as a major determining factor in the growth physiology.

The changes in pigment content as growth rates increased toward the upper bound of light-limitation, reflect the capacity of *Synechococcus* 7002 to acclimate phenotypically. Photoacclimation is a consequence of changes in gene expression and biosynthesis in response to changes in the light climate, and produces alterations in the macromolecular composition and architecture of the photosynthetic apparatus (Falkowski and Laroche, 1991; Durnford and Falkowski, 1997). The latter has been found to vary among organisms, and two classes of photoacclimation responses have been characterized (Falkowski and Owens, 1980). The number of photosynthetic reaction centers may be varied, with a relatively constant amount of pigment associated with each center. Alternatively, the number of reaction centers may remain constant, while large changes occur in the amount of phycobilin pigments transferring excitation energy to each reaction center. Because *Synechococcus* 7002 produces phycobilisomes with nearly invariant ratio of phycocyanin to allophycocyanin, changes in phycobiliproteins content as a function of light limitation must arise from changes in the number of phycobilisomes associated with thylakoids (de Marsac et al., 1988; Macintyre et al., 2002). There are also changes found in Chl associated with and proportional to the number of photosynthetic reaction centers, but these are more modest and suggest that phycobilisome number is being controlled to some degree.

The observed increase in the rETR_max_ with growth rate suggests an increase in number of photosynthetic units at faster growth rates, which also corresponds with increasing I_i_ during the light-limited conditions. This has also been observed for a number of other phototrophic microorganisms (Macintyre et al., 2002) and is consistent with the principle that the energy-generating machinery will be upregulated at higher growth rates. However, the decreased phycobiliprotein content in *Synechococcus* 7002 at higher growth rates and I_i_ values suggest that cells have fewer antennae complexes for light harvesting. This can help reduce photoinhibition effects by minimizing excess light absorption that can result in damage (Barber and Andersson, 1992).

Our measured changes in photosynthetic unit number and stoichiometry (Figure 6) can be related to analogous studies on *Synechocystis* PCC 6714 (Murakami et al., 1997) conducted using orange [preferentially absorbed by PS II associated physobilisomes (*sec*)], or red [absorbed mainly by Chl *a* and exciting mainly PS I (*sec*)] light. Notably, the PS II:PS I ratio in PCC 6714 was 2.6-fold lower in cells grown under orange (PS II) light than in those grown under red (PS I) light, which was similar to the current study. We believe that differences in PS II:PS I stoichiometry in both cyanobacterial strains are primarily due to the regulation of PS I expression levels. The current study found that the P700 content in *Synecococcus* 7002 cultures grown with 680-nm light was ~1.7-fold higher than in those grown only with 630-nm light. There was also a modest increase in PS I content of *Synechococcus* 7002 with growth rate for all light treatments, except for the case when fixed amount of 680-nm light was augmented with increasing amounts of 630 nm irradiance. Here, the high PS I contents found when only 680 nm was provided declined progressively to contents typically found in 630 nm-rich conditions.

Although a distinct phenotype can be observed in the absence of 630 nm irradiance, quanta that are specifically absorbed by the phycobilisomes (represented as 630 nm light) are not essential. Growth of *Synechococcus* 7002, albeit at slower growth rates (~50%), was also supported by monochromatic 680 nm irradiance. As phycobilisomes are thought to be primarily associated with PS II, its activity can be limiting here, as light is absorbed by chlorophyll *a* which is approximately three-fold more abundant in PS I than in PS II. The 680 nm light regime also corresponded to relatively higher photosynthetic potential, including: Y_II_, rETR_max_, PQ oxidation, CEF, and phycobiliprotein content. In terms of photosystem stoichiometry, the results in *Synechococcus* 7002 were very different from those reported for PCC 6714 (Murakami et al., 1997). Whereas the P700 content and PS II:PS I stoichiometry were highest in 7002 under monochromatic 680-nm irradiance, in PCC 6714 the P700 content and PS II:PS I ratio under “PS I light” were 50–57% of that found in cells grown with “PS II light.” Surprisingly, this implies that in order to sustain growth under monochromatic 680 nm illumination (albeit at reduced growth rates) higher levels of the photosystems were required than when both photosynthetic pigments could absorb radiation. Because the comparative increase in the photosynthetic performance parameters is evaluated as a function of growth rate, it is evident that the slower growth rate observed under monochromatic 680 nm illumination implies that a greater proportion of the resources acquired through photosynthesis must be used to produce the same amount of growth as can be produced with less input under dichromatic light conditions.

## Concluding remarks

Optically thin turbidostat photobioreactors were used to study the effects of growth rate on the physiology and photosynthetic performance of *Synechococcus* 7002, obtained during distinct mono- and dichromatic irradiance regimes. In general, the physiological parameters measured here increased with growth during dichromatic growth, regardless of the relative 630:680 nm ratio. Furthermore, if only 630 nm monochromatic light was provided, culture growth rates were similar to those obtained when the same total intensity of two wavelengths was used. We infer that these results reflect the plasticity of the photosynthetic apparatus to photoacclimate via a series of mechanisms including modulation of phycobilisome and reaction center size and stoichiometry, as well as the ability to redistribute excitation energy efficiently and effectively between the two photosystems.

### Conflict of interest statement

The authors declare that the research was conducted in the absence of any commercial or financial relationships that could be construed as a potential conflict of interest.
