# Supplementary material for: Effect of mono- and dichromatic light quality on growth rates and photosynthetic performance of Synechococcus sp. PCC 7002
Source: Front Microbiol. 2014 Sep 19;5:488. doi: 10.3389/fmicb.2014.00488 (PMC4168726; doi:10.3389/fmicb.2014.00488)
Supplement: Supplementary file 1 [file Presentation1.PDF]

Supplementary material

Effect of mono- and dichromatic light quality on growth rates and photosynthetic performance of *Synechococcus* sp. PCC 7002

Hans C. Bernstein[1,2], Allan Konopka[1,3], Matthew R. Melnicki[1], Eric A. Hill[1], Leo A. Kucek[1], Shuyi Zhang[4], Gaozhong Shen[3], Donald A. Bryant[4,5], and Alexander S. Beliaev[1*]

[1]Biological Sciences Division, Pacific Northwest National Laboratory, Richland, WA 99352; [2] Chemical and Biological Signature Science, Pacific Northwest National Laboratory, Richland, WA 99352; [3]Department of Biological Sciences, Purdue University, W. Lafayette, IN 47907; [4]Department of Biochemistry and Molecular Biology, The Pennsylvania State University, University Park, PA 16802; [5]Department of Chemistry and Biochemistry, Montana State University, Bozeman, MT 59717.

***Correspondence**:
Alexander S. Beliaev, Biological Sciences Division, Pacific Northwest National Laboratory, P. O. Box 999, MS: P7-50, Richland, WA 99352, phone: (509) 376-6966, fax: (509) 372-1632, email: alex.beliaev@pnnl.gov

1. PHOTOSYNTHEIC PARAMETERS AND RELATED DEFINITIONS

PS II: Photosystem II
PS I: Photosystem I
PQ: Plastoquinone pool
I: Scalar irradiance
F: Fluorescence yield assessed in conjunction with the application of a saturation pulse
Fm': Maximal fluorescence yield in illuminated samples upon application of a saturating pulse
Fv': Variable fluorescence in illuminated samples equated to (Fm'-F)
Y_{II}: Effective quantum yield of PS II and equated to Fv'/Fm'
rETR: Relative rate of electron transport (rate of charge separation at PS II) equated to ($I \cdot Y_{II}$)
OJIP-SMT: Variable Chl fluorescence transient corresponding to fluorescence induction (FI)
P-to-S: Normalized Difference between fluorescence at the P and S states (FP-FS)/(FP-FO)
CEF: Rate of cyclic electron flow measured as a post illumination rise in fluorescence
PQ-oxidation: Rate of dark PQ oxidation measured as a post illumination fluorescence decay

2. SUPPLEMENTARY METHODOLOGY

Determining chlorophyll and phycocyanin concentrations. A previously described "whole-cell spectra" method was used to determine the concentrations of *Chl* a and phycocyanin (Burns et al., 2006). Briefly, the absorbance's from whole-cell spectra were corrected for scatter with Equations S1 and S3; taken and modified from (Myers, 1980). The concentrations of Chl a and phycocyanin (nmol/ml) were calculated from the corresponding *corrected absorbance;* Equations S2 and S4, respectively.

$$A_{Phyco} = 1.0162 \cdot A_{630} - 0.2612 \cdot A_{680} \qquad \text{Eq. S1}$$

$$\frac{nmol\ phycocyanin}{ml} = \frac{A_{phyco} \cdot 1000}{111} \qquad \text{Eq. S2}$$

$$A_{Chl} = 1.0162 \cdot A_{680} - 0.063 \cdot A_{630} \qquad \text{Eq. S3}$$

$$\frac{nmol\ Chl\ A}{ml} = \frac{A_{Chl} \cdot 893}{68} \qquad \text{Eq. S4}$$

3. SUPPLEMENTARY SUPPORTING DATA

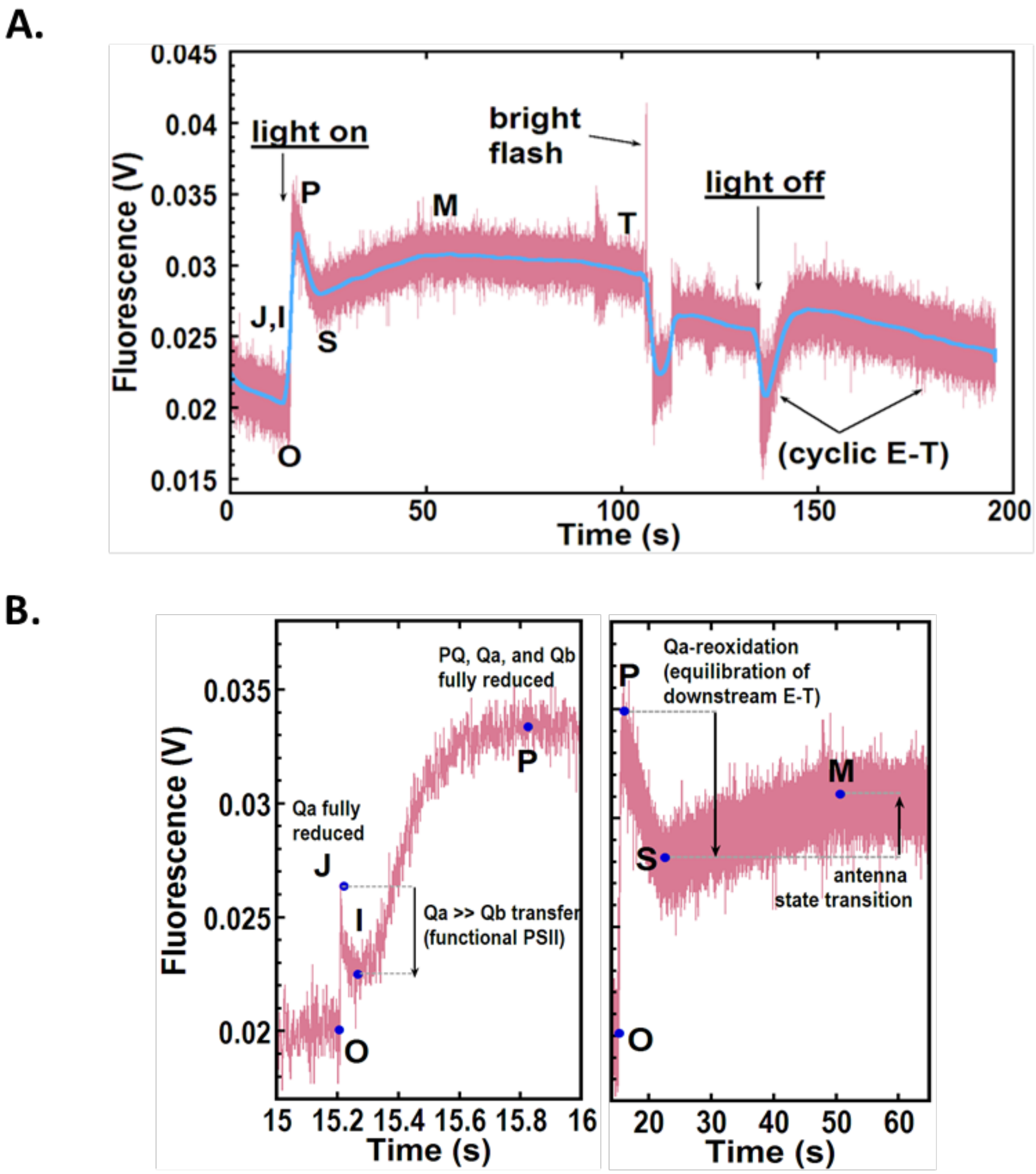

Figure S1. Example fluorescence transient trace highlighting the OJIP-SMT light induction kinetics and post variable chlorophyll fluorescence analyses used in this study. (A) Entire light induction fluorescence trace and (B) truncated OJIP-SM transients. The abreviations for OJIP-SMT are as follows: (O) origin and first measured minimum fluorescence level., (J and I) intermediate inflections between O and (P) peak, (S) semi-steady state fluorescence level, (M) maximum fluorescence and (T) Terminal) steady-state level.

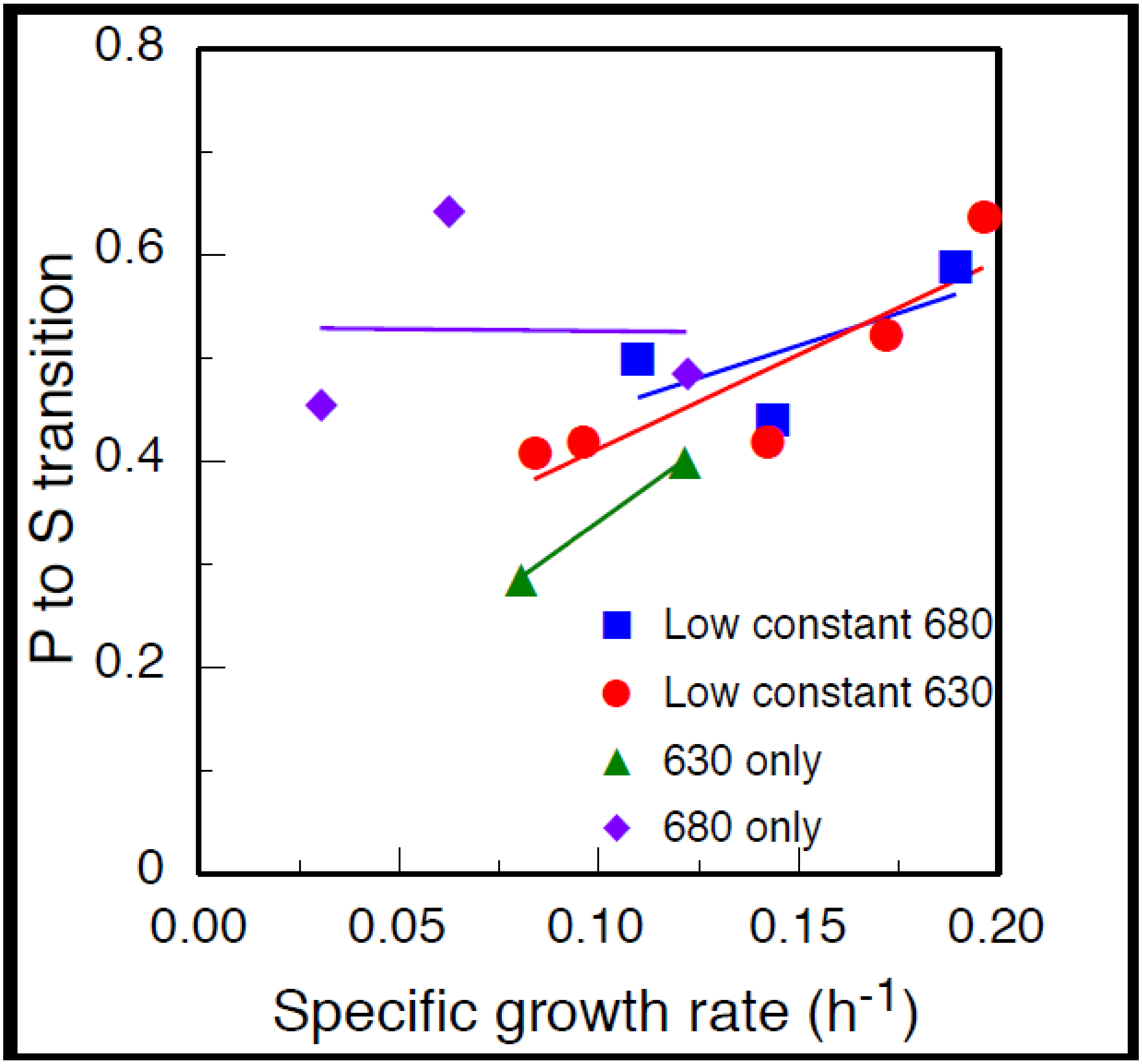

Figure S2. P-to-S transition data for P>>S representing the quenching of Chl fluorescence from the maximally reduced PQ-pool state ("P") to the quasi steady-state ("S") state (ca. 16 to 24 sec) during assay of Chl fluorescence induction kinetics used in this study. Measurements correspond to samples adapted at four distinct light regimes each corresponding to mono- or dichromatic irradiance imposed in the steady state turbidostat: (■) 53 µmol m^{-2} s^{-1} of 680 nm light plus variable amounts of 630 nm light, (●) 50 µmol m^{-2} s^{-1} of 630 nm light plus variable amounts of 680 nm light, (▲) 630 nm light only, or (♦) 680 nm light only.

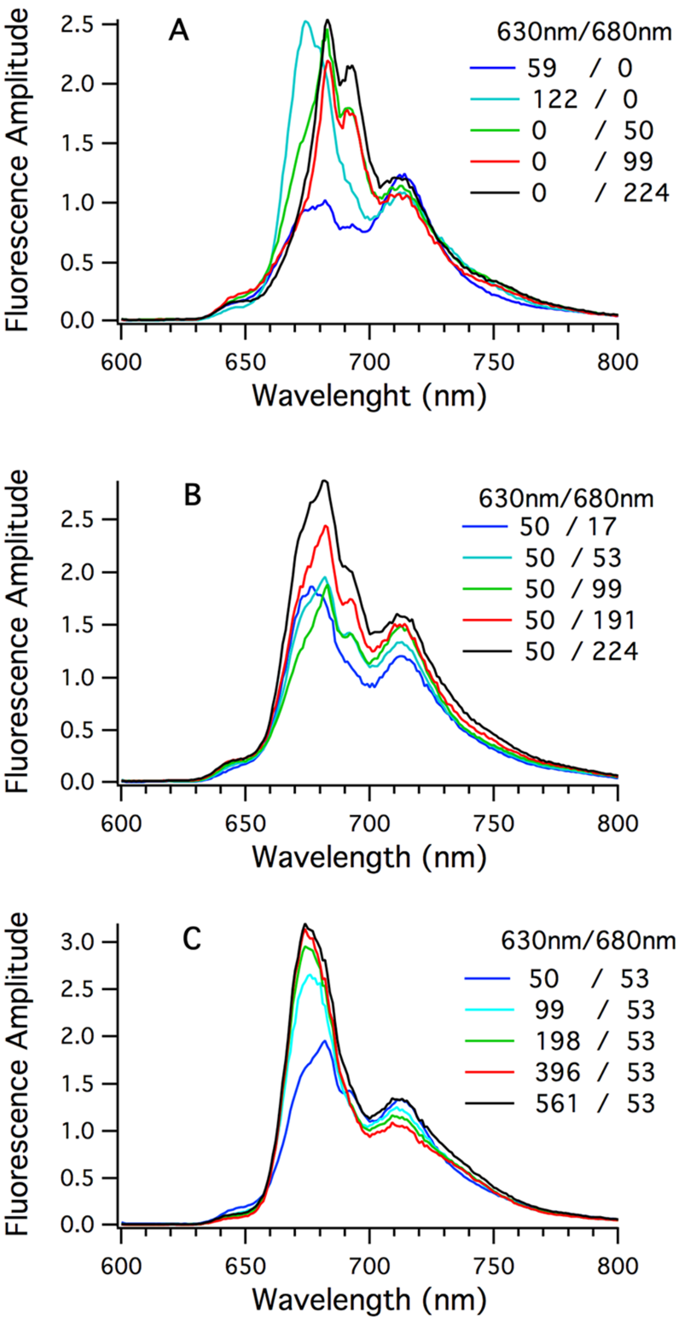

Figure S3. The low-temperature fluorescence emission spectra of *Synechococcus* sp. PCC 7002 cells grown at different light conditions. A), cells grown at either LED630nm or LED680nm light conditions with variation of light intensities; B), cells grown at different intensities of LED680nm light with consistent LED630nm light at 50 µE.m^{-2}.s^{-1}; and C), cells grown at different intensities of LED630nm light with consistent LED680nm light at 53 µE.m^{-2}.s^{-1}. Cells were adjusted to $OD_{730\ nm}$=0.5 in 50 mM HEPES, pH=7 with 60% glycerol before quickly frozen in liquid nitrogen. The excitation wavelength was 440nm for mostly exciting chlorophylls. Each spectrum was average of four measured spectra.

Table 1. Coefficients of determination from linear regression of data provided in main figures.

Figure #	Data Set*	Coefficient (slope)	Standard Error	Lower 90% Confidence Limit	Upper 90% Confidence Limit	R^2
Fig. 1A	C680	0.00052	0.00005	-0.00011	0.00116	0.981
Fig. 1A	C630	0.00053	0.00005	0.00036	0.0007	0.960
Fig. 1A	680 only	0.00052	0.00004	0.00005	0.00099	0.990
Fig. 1B	C680	74.36	3.24	33.19	115.53	0.996
Fig. 1B	C630	110.93	8.10	85.14	136.74	0.979
Fig. 1B	680 only	40.50	0.41	35.26	45.75	0.999
Fig. 3A	C680	0.41	0.32	-1.63	2.45	0.560
Fig. 3A	C630	1.00	0.16	0.61	1.39	0.927
Fig. 3A	680 only	0.60	0.45	-2.24	3.44	0.658
Fig. 3B	C680	25.51	20.86	-106.16	157.19	0.599
Fig. 3B	C630	100.29	10.80	74.87	125.71	0.966
Fig. 3B	680 only	73.80	2.47	58.19	89.43	0.997
Fig. 4A	C680	0.00062	0.00042	-0.0020	0.00329	0.685
Fig. 4A	C630	0.00034	0.00011	0.000085	0.00059	0.768
Fig. 4A	680 only	-0.00010	0.00028	-0.0019	0.0017	0.121
Fig. 4B	C680	0.00686	0.0027	-0.010	0.024	0.864
Fig. 4B	C630	0.010	0.0029	0.0033	0.016	0.803
Fig. 4B	680 only	0.00036	0.0068	-0.043	0.043	0.002
Fig. 5A	C680	-0.317	0.18	-1.50	0.870	0.740
Fig. 5A	C630	-0.185	0.024	-0.254	-0.114	0.967
Fig. 5A	680 only	-0.138	0.166	-1.18	0.909	0.409
Fig. 5B	C680	-0.0349	0.070	-0.476	0.407	0.199
Fig. 5B	C630	-0.080	0.0019	-0.086	-0.074	0.999
Fig. 5B	680 only	-0.050	0.051	-0.37	0.27	0.495
Fig. 6A	C680	0.17	0.65	-3.91	4.25	0.064
Fig. 6A	C630	2.94	0.73	1.23	4.65	0.845
Fig. 6A	680 only	1.62	1.00	-4.71	7.94	0.722
Fig. 6B	C680	-995.50	152.43	-1957.86	-33.09	0.977
Fig. 6B	C630	979.15	573.51	-370.53	2328.82	0.49
Fig. 6B	680 only	380.83	359.18	-1886.92	2648.60	0.529

* C denotes wavelength held constant

4. SUPPLEMENTARY REFERENCES CITED

Burns, R.A., Mac Kenzie, T.D., and Campbell, D.A. (2006). Inorganic carbon repletion constrains steady-state light acclimation in the cyanobacterium *Synechococcus elongatus*. *Journal of Phycology* 42**,** 610-621.

Myers, J. (1980). "On the algae: thoughts about physiology and measurements of efficiency," in *Primary productivity in the sea*. Springer), 1-16.
